# Immunocytochemical Analysis of Bifid Trichomes in *Aldrovanda vesiculosa* L. Traps

**DOI:** 10.3390/ijms24043358

**Published:** 2023-02-08

**Authors:** Bartosz J. Płachno, Małgorzata Kapusta, Piotr Stolarczyk, Magdalena Wójciak, Piotr Świątek

**Affiliations:** 1 Department of Plant Cytology and Embryology, Institute of Botany, Faculty of Biology, Jagiellonian University, 9 Gronostajowa St., 30-387 Kraków, Poland; 2 Department of Plant Cytology and Embryology, Faculty of Biology, University of Gdańsk, 59 Wita Stwosza St., 80-308 Gdańsk, Poland; 3 Department of Botany, Physiology and Plant Protection, Faculty of Biotechnology and Horticulture, University of Agriculture in Kraków, 29 Listopada 54 Ave., 31-425 Kraków, Poland; 4 Department of Analytical Chemistry, Medical University of Lublin, Chodźki 4a, 20-093 Lublin, Poland; 5 Institute of Biology, Biotechnology and Environmental Protection, Faculty of Natural Sciences, University of Silesia in Katowice, 9 Bankowa St., 40-007 Katowice, Poland

**Keywords:** aquatic plant, arabinogalactan proteins, carnivorous plants, cell wall, *Aldrovanda*, Droseraceae, mucilage trichomes, transfer cells, wall ingrowths

## Abstract

The two-armed bifids (bifid trichomes) occur on the external (abaxial) trap surface, petiole, and stem of the aquatic carnivorous plant *Aldrovanda vesiculosa* (Droseracee). These trichomes play the role of mucilage trichomes. This study aimed to fill the gap in the literature concerning the immunocytochemistry of the bifid trichomes and compare them with digestive trichomes. Light and electron microscopy was used to show the trichome structure. Fluorescence microscopy revealed the localization of carbohydrate epitopes associated with the major cell wall polysaccharides and glycoproteins. The stalk cells and the basal cells of the trichomes were differentiated as endodermal cells. Cell wall ingrowths occurred in all cell types of the bifid trichomes. Trichome cells differed in the composition of their cell walls. The cell walls of the head cells and stalk cells were enriched with arabinogalactan proteins (AGPs); however, they were generally poor in both low- and highly-esterified homogalacturonans (HGs). The cell walls in the trichome cells were rich in hemicelluloses: xyloglucan and galactoxyloglucan. The cell wall ingrowths in the basal cells were significantly enriched with hemicelluloses. The presence of endodermal cells and transfer cells supports the idea that bifid trichomes actively transport solutes, which are polysaccharide in nature. The presence of AGPs (which are considered plant signaling molecules) in the cell walls in these trichome cells indicates the active and important role of these trichomes in plant function. Future research should focus on the question of how the molecular architecture of trap cell walls changes in cells during trap development and prey capture and digestion in *A. vesiculosa* and other carnivorous plants.

## 1. Introduction

Plant carnivory is a specialization for living in environments with limited macroelements [1,2], including shallow dystrophic waters [3,4]. Aquatic (submerged aquatic or amphibious) carnivorous plants comprise around 60 species of *Utricularia* L. and *Aldrovanda vesiculosa* L. [4]. *A. vesiculosa* (waterwheel plant) is a rootless, free-floating plant that occurs in all continents of the Old World and across various climatic zones; however, it is rare and endangered [5,6,7]. It is the phylogenetic sister of the Venus flytrap (*Dionaea muscipula* Ellis) [8], and the phylogenetic age of the genus *Aldrovanda* is about 48–53.4 Mya [9]. Its leaf terminates into a carnivorous snapping trap, with bristles for catching small invertebrates [10,11]. The *Aldrovanda* trap is a delicate organ, 2–4 mm long, consisting of two lobes connected via a midrib. *Aldrovanda* traps perform one of the fastest movements known in the plant kingdom because the trap closes within ~10–100 ms after mechanical triggering by prey [12]. Only *Utricularia* species can compete with *A. vesiculosa*, the traps of which are even faster [13]. On the inner and outer surface of *A. vesiculosa* traps, there is a variety of trichomes, which perform various functions: two-armed trichomes (bifids), four-armed trichomes (cruciform trichomes or quadrifids), bun-shaped trichomes (digestive and absorptive glands), and sensitive trichomes [10]. According to Lloyd [10], both bifids and quadrifid trichomes secrete mucilage. Koller-Peroutka et al. [14] used X-ray microanalysis to compare element contents and distributions in the glands of two aquatic species, *Utricularia purpurea* and *A. vesiculosa*. The elemental composition of the quadrifid trichomes and the digestive trichomes was clearly different from the bifids. Thus, these authors proposed that this result reflects the different functions of the various trichome types. Most researchers have focused on the digestive trichomes of *A. vesiculosa*. The dynamic changes of the *A. vesiculosa* digestive trichome ultrastructure have been described [15,16,17]. Adlassnig et al. [18] experimentally proved that in these trichomes, nutrient uptake also occurs by endocytosis. Recently, we studied the carbohydrate epitopes associated with the major cell wall polysaccharides and the glycoproteins in the digestive glands of *A. vesiculosa*. and *D. muscipula* [19,20]. In both species, in the digestive gland cells, the wall ingrowths were rich in arabinogalactan proteins, which probably play a signaling role, especially because the glands are the most physiologically active part of the traps.

Both *A. vesiculosa* and *D. muscipula* have snapping-type traps, which shut after the reception of an external mechanical stimulus from animals. In addition, both use jasmonates to activate a carnivorous response [21,22]. However, according to Poppinga and Joyeux [23], these species have entirely different closure mechanisms: in *Dionaea*, closing comprises abrupt curvature inversion of the two trap lobes. In contrast, the closing movement in *Aldrovanda* involves deformation of the trap midrib but not the lobes, which do not change curvature. Both *A. vesiculosa* and *D. muscipula* have external (abaxial) trap trichomes: bifids in *A. vesiculosa* and star-shaped stellate trichomes in *D. muscipula*. Recently, we studied the carbohydrate epitopes associated with the major cell wall polysaccharides and the glycoproteins in the stellate trichomes [24], so our first aim was to compare them with the bifids in *A. vesiculosa*. *D. muscipula’s* traps mainly act in a terrestrial environment, while *A. vesiculosa* catches aquatic prey; thus, it would be interesting to evaluate the differences between the trichomes in these species. The second aim was to show the different characteristics among the variable types of trichome of *A. vesiculosa*.

## 2. Results

### 2.1. Trichome Structure

**Figure 1:**
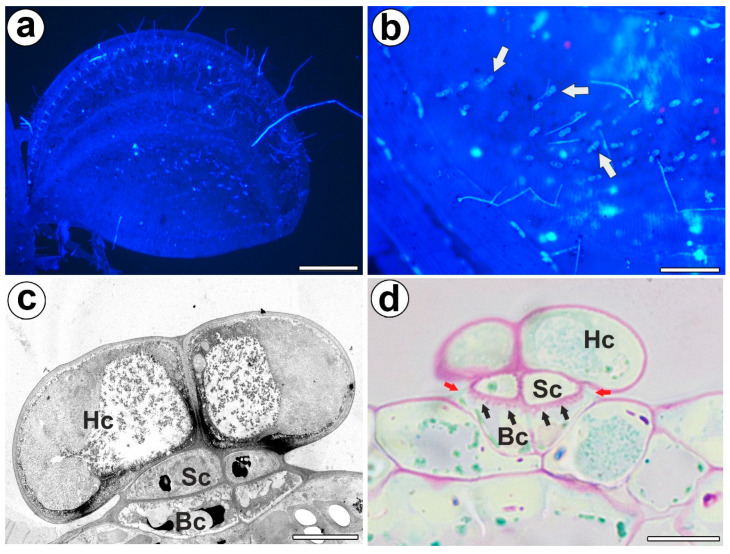
Distribution, morphology, and structure of the bifid trichomes of the *Aldrovanda vesiculosa* traps. (**a**) *Aldrovanda vesiculosa* trap, bar 500 µm. (**b**) Bifid trichomes on the external surface of a trap (arrow), bar 100 µm. (**c**) Ultrastructure of a bifid trichome; head cell (Hc), stalk cell (Sc), basal cell (Bc), bar 500 nm. (**d**) Positive result of the PAS reaction of the cell wall ingrowths (black arrows); head cell (Hc), stalk cell (Sc), basal cell (Bc), Casparian strip (red arrows), bar 10 µm.

Bifid trichomes were present on the trap petiole, the outer part of the trap lobes (Figure 1a,b), and at the stem. Each bifid trichome consisted of two basal cells, two stalk cells, and a head with two cells (Figure 1c). The outer lateral cell walls of the stalk cells and basal cells were cutinized, and thus they formed the Casparian strip (Figure 1d). The outer lateral cell walls of the basal cell were especially strongly cutinized, which was seen after PAS reaction (lipophilic substance inhibits access to pectins) (Figure 1d). Thus, both the stalk and basal cells performed a function as endodermal cells. PAS reaction showed well-developed cell wall ingrowths in the basal cells (Figure 1d). Cell wall ingrowths also occurred in the stalk cells and head cells. In the cytoplasm of the head cell, there were active dictyosomes with vesicles.

### 2.2. The Arabinogalactan Protein (AGPs) Distribution

**Figure 2:**
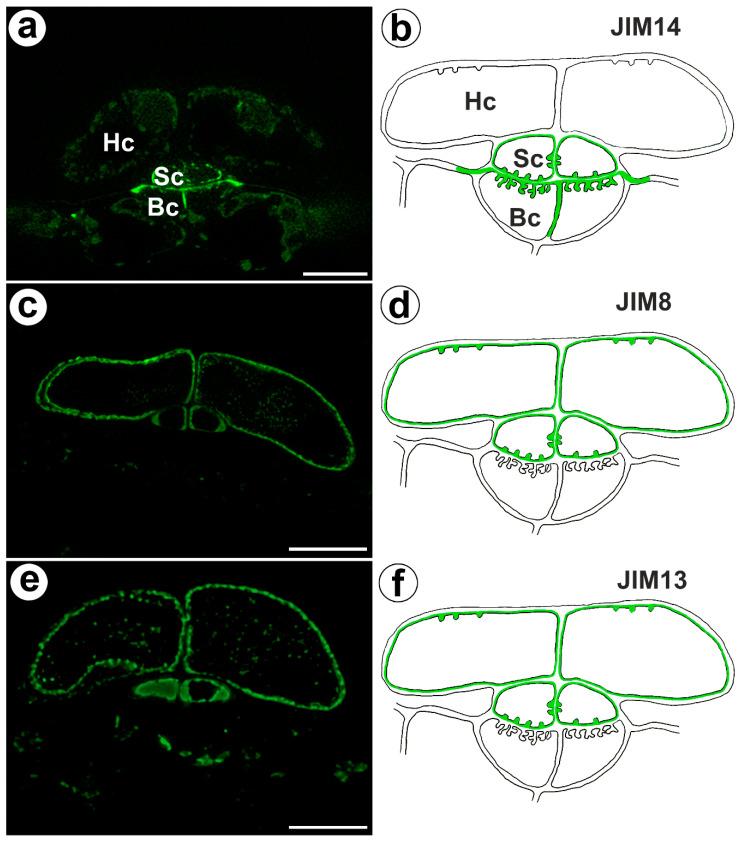
Arabinogalactan proteins detected in the bifid trichomes of the *Aldrovanda vesiculosa* traps. (**a**) Arabinogalactan proteins (labeled with JIM14) were detected in the trichomes, head cell (Hc), stalk cell (Sc), basal cell (Bc), and bar 10 µm. (**b**) Schematic occurrence (green) of the arabinogalactan proteins (labeled with JIM14) detected in trichome, head cell (Hc), stalk cell (Sc), and basal cell (Bc). (**c**) Arabinogalactan proteins (labeled with JIM8) were detected in trichomes, bar 10 µm. (**d**) Schematic occurrence (green) of the arabinogalactan proteins (labeled with JIM8) detected in a trichome. (**e**) Arabinogalactan proteins (labeled with JIM13) detected in trichomes, bar 10 µm. (**f**) Schematic occurrence (green color) of the arabinogalactan proteins (labeled with JIM13) detected in a trichome.

**Figure 3:**
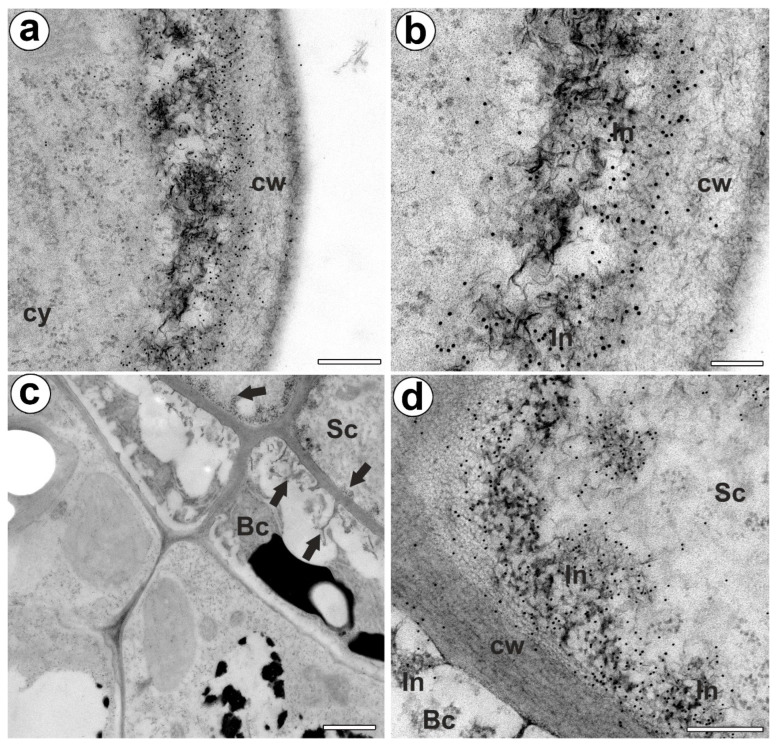
Arabinogalactan proteins (labeled with JIM13) detected in the bifid trichomes of the *Aldrovanda vesiculosa* traps. (**a**,**b**) Immunogold labeling of wall ingrowths with JIM13 in head cell; cytoplasm (cy), cell wall (cw), cell wall ingrowth (In), bar 300 nm, and bar 100 nm. (**c**) Immunogold labeling of cell walls with JIM13 in stalk (Sc) and basal cells (Bc), cell wall ingrowths (arrows), bar 1000 nm. (**d**) Immunogold labeling of cell wall ingrowths with JIM13 in stalk and basal cells; stalk cell (Sc), basal cell (Bc) cell wall ingrowth (In), bar 300 nm.

The epitope recognized by JIM14 occurred in the cell walls of the stalk cells and basal cells (in the place where cell wall ingrowths did occur) (Figure 2a,b). The AGP epitope recognized by the JIM8 antibody was present in the head cells and in the cell walls of the stalk cells (Figure 2c,d). The AGP epitope that is recognized by JIM13 was mainly present in the cell walls of the head cells and stalk cells (Figure 2e,f). The immunogold labeling with JIM13 showed that the AGP epitopes were localized in the cell walls, and the wall ingrowths in the head cells (Figure 3a,b). Gold particles were abundant in the wall ingrowths in the stalk cells (Figure 3c,d). Gold particles were less abundant near the cell wall ingrowths in the basal cells (Figure 3c,d).

### 2.3. Homogalacturonan Distribution

**Figure 4:**
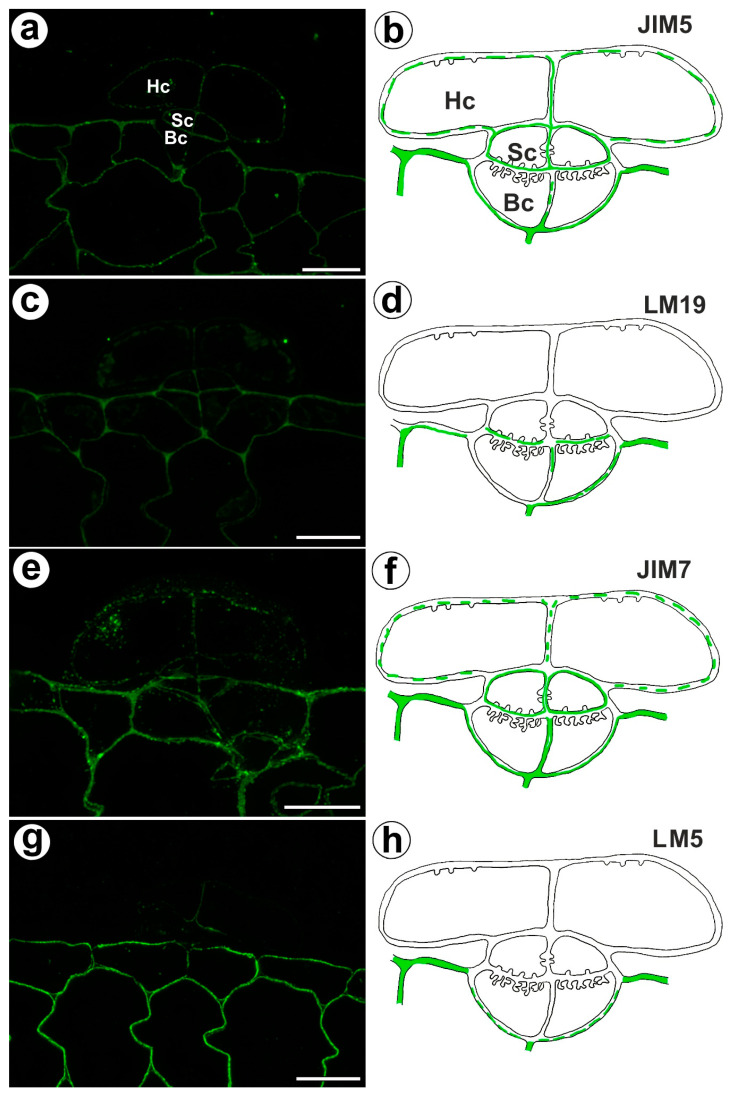
Homogalacturonan detected in the bifid trichomes of the *Aldrovanda vesiculosa* traps. (**a**) HG (labeled with JIM5) that was detected in a trichome, head cell (Hc), stalk cell (Sc), basal cell (Bc), bar 10 µm. (**b**) Schematic occurrence (green) of the HG (labeled with JIM5) detected in a trichome, head cell (Hc), stalk cell (Sc), basal cell (Bc). (**c**) HG (labeled with LM19) detected in a trichome, bar 10 µm. (**d**) Schematic occurrence (green) of the HG (labeled with LM19) detected in a trichome. (**e**) HG (labeled with JIM7) detected in a trichome, bar 10 µm. (**f**) Schematic occurrence (green) of the HG (labeled with JIM7) found in a young trichome. (**g**) HG (labeled with LM5) detected in a trichome, bar 10 µm. (**h**) Schematic occurrence (green) of the HG (labeled with LM5) detected in a trichome.

The fluorescence signal detected by JIM5 (low methyl esterified HGs) was observed in a trichome in the cell walls of the basal cells, stalk cells, and head cells, as well as in the cell walls in ordinary epidermal cells and parenchyma cells of the traps (Figure 4a,b). The fluorescence signal was more intensive in the ordinary epidermal cells and parenchyma cells of traps than in the trichome cells. In the trichome, the weak fluorescence signal detected by LM19 (low methyl esterified HGs) was not observed in the head cell walls but did occur in the cell walls of basal cells and the cell walls in ordinary epidermal cells and parenchyma cells (Figure 4c,d). An intense fluorescence signal from highly esterified HGs (detected by JIM7) was observed in the cell walls of the epidermal and parenchyma cells of the traps (Figure 4e,f). A delicate signal of this epitope was observed in the cell walls of the trichome cells (Figure 4e,f). An intense signal from the pectic polysaccharide (1–4)-β-D-galactan (detected by LM5) was observed in the cell walls of the epidermal and parenchyma cells of the traps (Figure 4g,h) but did not occur in trichome cells or only slightly occurred in the head cells (Figure 4g,h).

### 2.4. Hemicellulose Distribution

**Figure 5:**
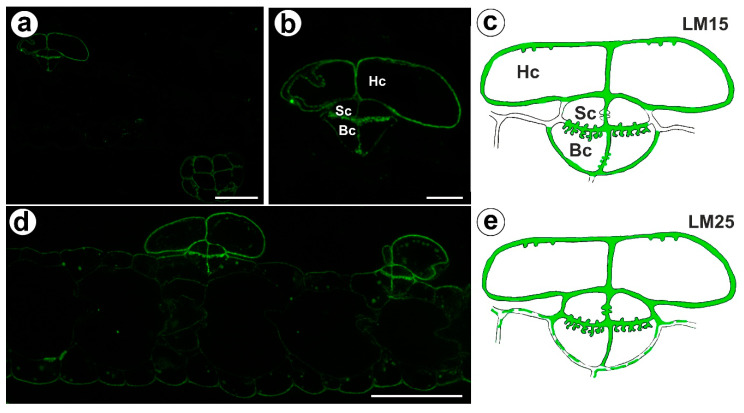
Xyloglucan detected in the bifid trichomes of the *Aldrovanda vesiculosa* traps. (**a**) Xyloglucan (labeled with LM15) detected in a trichomes and trap wall, bar 25 µm. (**b**) Xyloglucan (labeled with LM15) detected in a trichome, head cell (Hc), stalk cell (Sc), basal cell (Bc), bar 10 µm. (**c**) Schematic occurrence (green) of the xyloglucan (labeled with LM15) detected in a trichome; head cell (Hc), stalk cell (Sc), basal cell (Bc). (**d**) Xyloglucan (labeled with LM25) detected in a trichome, bar 25 µm. (**e**) Schematic occurrence (green) of the xyloglucan (labeled with LM25) detected in a trichome.

The signal from xyloglucan (detected by LM15) was observed in the cell walls of the basal cells, the stalk cells, and the head cells (Figure 5a,c). A very intense fluorescence signal from this xyloglucan was observed in the basal cells in the cell wall ingrowths (Figure 5b,c). Xyloglucan epitopes (detected by LM25) occurred in the cell walls in all cells of the trichomes (Figure 5d,e). A very intense fluorescence signal from this xyloglucan was observed in the basal cells in the cell wall ingrowths (Figure 5d,e). A weak fluorescence signal from this xyloglucan was observed in the ordinary epidermal cells and parenchyma cells (Figure 5d,e).

## 3. Discussion

*Dionaea muscipula* and *Aldrovanda vesiculosa* have a similar type of trap; however, they differ in their outer trichomes. *Aldrovanda’s* bifid trichomes have a simpler structure than *Dionaea muscipula’s* stellate trichomes, consisting of a smaller number of cells. *A. vesiculosa* has two-celled heads in the bifid trichome. The head of stellate trichome in *D. muscipula* consists of two “internal head cells” and up to eight elongated outer head cells [10]. Both types of trichomes produce polysaccharides (mucilage). In *Aldrovanda*, polysaccharides are produced by bifids in mature traps (however, there is a lack of research on whether mucilage production already occurs in immature traps); however, in *D. muscipula*, stellate trichomes produce mucilage in immature traps. In mature traps, stellate trichome changes their function (their outer head cells die) [20]. Ivanova and Muravnik [25] suggested that bifids play a role in *Aldrovanda* trap reopening by producing osmotically active compounds that make water inflow.

AGPs are widespread in plant cells [26] and play various essential roles in plants: cell wall expansion, cell differentiation, tissue development, calcium capacitors, and somatic embryogenesis [27,28], as well as plant reproduction [29,30,31,32]. Our current results confirm previous results where AGPs in carnivorous plant traps were mainly found in glandular structures (trichomes, glands): digestive glands of *Dionaea muscipula* [20], and *Aldrovanda* [19], stellate trichomes of *D. muscipula* [24]. AGPs (JIM13) were also recorded in *Drosera capensis* glands by Samaj et al. [33]. The occurrence of AGPs is associated with glandular cells and/or the presence of cell wall ingrowths. Especially in Droseraceae, cell wall ingrowths may also occur in the basal and stalk cells of trichomes. It should be noted that the occurrence of an individual type of arabinogalactan protein varies between different transfer cell types in the bifid trichomes. The cell walls of the head and stalk cells were enriched with arabinogalactan proteins (JIM8 and JIM13), but at the light microscopy level, we could not record these AGPs in the cell walls in basal cells. However, using electron microscopy through immunogold labeling with JIM13, we showed that the AGP epitopes occur in basal cells. There is variability in the occurrence, abundance, and type of polymers in the cell wall ingrowths among angiosperms and bryophytes [34,35,36,37,38]. It was proven that AGPs are involved in vesicle trafficking; AGP excretion occurs via exocytosis [33,39]. According to McCurdy et al. [40], AGPs participate in coordinating the required localized assembly of wall components; thus, they play a role in forming cell wall ingrowths. According to Olmos et al. [39], AGP accumulation might regulate the cell wall extensibility, thus affecting the pectin network as plasticizers and facilitating cell expansion. However, AGPs could also participate in the signaling pathways in plants. Thus, the presence of AGPs in bifids may be related to both the presence of transfer cells and the secretory role of these trichomes. Moreover, if Ivanova and Muravnik [25] are correct about the function of bifids, then AGPs could also participate in the process of opening traps.

HGs are involved in plant cell wall porosity, elasticity, hydration, and cellular adhesion/separation [41,42]. Traps in Droseraceae are transformed leaves, so it is not surprising that the epidermal cells and parenchyma cell walls in both *Aldrovanda* and *D. muscipula* are rich in HGs, as in the leaves of other plants [43]. In bifids, the cell walls of the outer head cells were poor in both low and highly-esterified HGs, which is very similar to the occurrence of these HGs in stellate trichomes in *D. muscipula* [24]. The difference is in the presence of galactan. When stellate trichomes produce mucilage, the cell walls of the head cell are rich in it. However, when the stellate trichomes were fully differentiated, this galactan was lost in the thick walls of the outer terminal cells [24]. In bifids, we did not detect galactan in the cell walls of head cells.

Our work demonstrated that the hemicelluloses recognized by the LM25 antibodies (for galactoxyloglucan) and LM15 antibodies (for xyloglucan) were present in the cell walls in all bifid trichome cells. Xyloglucans are one of the most abundant hemicelluloses of the primary cell walls in dicots, where they play an important function in tethering the cellulose microfibrils together. The hemicellulose network maintains the strength of the primary cell walls and participates in their expansion [44,45,46,47]. We show that cell wall ingrowths in the basal cell of bifids are enriched with xyloglucans. These xyloglucans were also recorded in the cell walls and cell wall ingrowths in the transfer cells of *A. vesiculosa* digestive glands [19]. Galactoxyloglucan occurs in the cells of stellate trichomes of *D. muscipula*. Nevertheless, xyloglucan (LM15) was lacking in cell walls in the outer terminal cells of these trichomes [24]. Xyloglucan (LM15) and galactoxyloglucan (LM25) were also recorded in the cell wall ingrowths in bryophytes [38]. Xyloglucans were also localized in cell wall ingrowths in epidermal transfer cells of *Vicia faba* cotyledons [34]. According to Henry and Renzaglia [38], xyloglucans in transfer cell walls are a regulator of cell wall extensibility, by weakening the cellulose network, to allow slippage during cell growth. We observed a signal from galactoxyloglucan (LM25) in the cell walls of parenchyma and epidermis of traps; however, a signal from xyloglucan (detected by LM15) was absent in these cells. The cell walls of parenchyma and epidermis of traps are rich in pectins, which may mask the LM15 xyloglucan epitope [47]. Hemicelluloses probably play a role as signaling molecules, e.g., in sexual plant reproduction [48,49,50]. Therefore, it cannot be ruled out that hemicellulose presence in transfer cells is also linked to such a role.

## 4. Materials and Methods

### 4.1. Plant Material

The *A. vesiculosa* L. plants (Polish clone) were collected from Mr. Maciej Kosiedowski’s (Tarnowskie Góry, Poland) private collection. For the bifid trichome analysis, mature traps were taken from mature plants at the same stage of development.

### 4.2. Histological and Immunochemical Analysis

The traps were fixed in 8% (*w*/*v*) paraformaldehyde (PFA, Sigma-Aldrich, Sigma-Aldrich Sp. z o.o. Poznań, Poland) and 0.25% (*w*/*v*) glutaraldehyde (GA, Sigma-Aldrich, Sigma-Aldrich Sp. z o.o. Poznań, Poland) in PIPES buffer overnight at 4 °C. The PIPES buffer contained 50 mM PIPES (piperazine-N,N′-bis [2-ethanesulfonic acid], Sigma-Aldrich, Sigma-Aldrich Sp. z o.o. Poznań, Poland), 10 mM EGTA (ethylene gly-col-bis[β-aminoethyl ether]N,N,N′,N′-tetraacetic acid, Sigma Aldrich, Poznań, Poland), and 1 mM MgCl_2_ (Sigma-Aldrich, Sigma-Aldrich Sp. z o.o. Poznań, Poland), pH 6.8. For the analysis of the occurrence of the major cell wall polysaccharides and glycoproteins, the plant material was embedded in LR White Resin (Polysciences Europe GmbH, Hirschberg a der Bergstrasse, Germany), which was repeated twice and then sectioned. The rehydrated sections were blocked with 1% bovine serum albumin (BSA, Sigma-Aldrich, Poznań, Poland) in a PBS buffer and incubated with the following primary antibodies: anti-AGP: JIM8, JIM13, and JIM14 [51,52,53,54,55], anti-pectin: JIM5, JIM7, LM19, LM5 [47,51,55,56]; and anti-hemicelluloses: LM25, LM15 [47,55,56] overnight at 4 °C. All of the primary antibodies were used in a 1:20 dilution. They were purchased from Plant Probes, UK, and the goat anti-rat secondary antibody conjugated with FITC was purchased from Abcam (Abcam plc, Cambridge, UK). The chromatin in the nuclei was stained with 7 µg/mL DAPI (Sigma-Aldrich, Sigma-Aldrich Sp. z o.o. Poznań, Poland) diluted in a PBS buffer. The samples were then cover-slipped using a Mowiol mounting medium: a mixture of Mowiol ^®^4-88 (Sigma-Aldrich, Sigma-Aldrich Sp. z o.o. Poznań, Poland) and glycerol for fluorescence microscopy (Merck, Warszawa, Poland) with the addition of 2.5% DABCO (The Carl Roth GmbH + Co. KG, Karlsruhe, Germany). They were viewed using a Nikon Eclipse E800 microscope or a Leica DM6000B microscope. Photos were acquired as Z stacks and deconvolved using five iterations of a 3D nonblind algorithm (AutoQuant™, Media Cybernetics Inc., Rockville, MD, USA). In order to maximize the spatial resolution, the images are presented as maximum projections. The stacks were obtained using a Leica DM6000B microscope equipped with a GFP filter. At least two different replications were performed for each of the analyzed traps, and about five to ten sections from each organ were analyzed for each antibody used. Negative controls were created by omitting the primary antibody step, which caused no fluorescence signal in any of the control frames for any stained slides (Figure S1). The immunogold procedure was performed as in Płachno et al. [19].

### 4.3. Light Microscopy (LM)

Semi-thin sections (0.9–1.0 µm thick) were prepared for LM and stained for the general histology using aqueous methylene blue/azure II (MB/AII) for 1–2 min [57]. The periodic acid-Schiff (PAS) reaction was also used to reveal the presence of any insoluble polysaccharides [58].

### 4.4. Transmission Electron Microscopy

The traps were also examined using transmission electron microscopy (TEM), as follows: Fragments of the traps were fixed in a mixture of 2.5% glutaraldehyde with 2.5% formaldehyde in a 0.05 M cacodylate buffer (Sigma-Aldrich, Sigma-Aldrich Sp. z o.o. Poznań, Poland; pH 7.2) overnight or for several days, washed three times in a 0.1 M sodium cacodylate buffer, and post-fixed in a 1% osmium tetroxide solution at room temperature for 1.5 h. This was followed by dehydration using a graded ethanol series, infiltration, and embedding using an epoxy embedding medium kit (Fluka). Following polymerization at 60 °C, sections were cut at 70 nm for the transmission electron microscopy (TEM) using a Leica ultracut UCT ultramicrotome, stained with uranyl acetate and lead citrate [59] and visualized using a Jeol JEM 100 SX microscope (JEOL, Tokyo, Japan) at 80 kV in the Department of Cell Biology and Imaging, Institute of Zoology, Jagiellonian University in Kraków or with a Hitachi UHR FE-SEM SU 8010 microscope at 25 kV, which is housed at the University of Silesia in Katowice.

## 5. Conclusions

Our cytological study indicates that bifid trichomes in *Aldrovanda* share features with stellate trichomes of *Dionaea*: the presence of transfer cells, accumulation of AGPs in cell walls, and the absence or only small amounts of HGs in the cell walls of trichome heads. Here, we show that AGPs occur not only in the digestive glands but also in other glands (glandular trichomes) in carnivorous plant traps. Future research should focus on the question of how the molecular architecture of trap cell walls changes in cells during trap development and in prey capture and digestion in carnivorous plants.

## Data Availability

The data presented in this study are available on request from the corresponding author.

## Supplementary Materials

The following supporting information can be downloaded at: https://www.mdpi.com/article/10.3390/ijms24043358/s1.
