# Supplementary material for: Immunocytochemical Analysis of Bifid Trichomes in Aldrovanda vesiculosa L. Traps
Source: Int J Mol Sci. 2023 Feb 8;24(4):3358. doi: 10.3390/ijms24043358 (PMC9958864; doi:10.3390/ijms24043358)

Supplementary material Figure S1. Control reaction of the immunolabeling. (**a**) Control reaction of the immunolabeling of the cell wall components that were detected in a mature trichome, bar 20 μm.

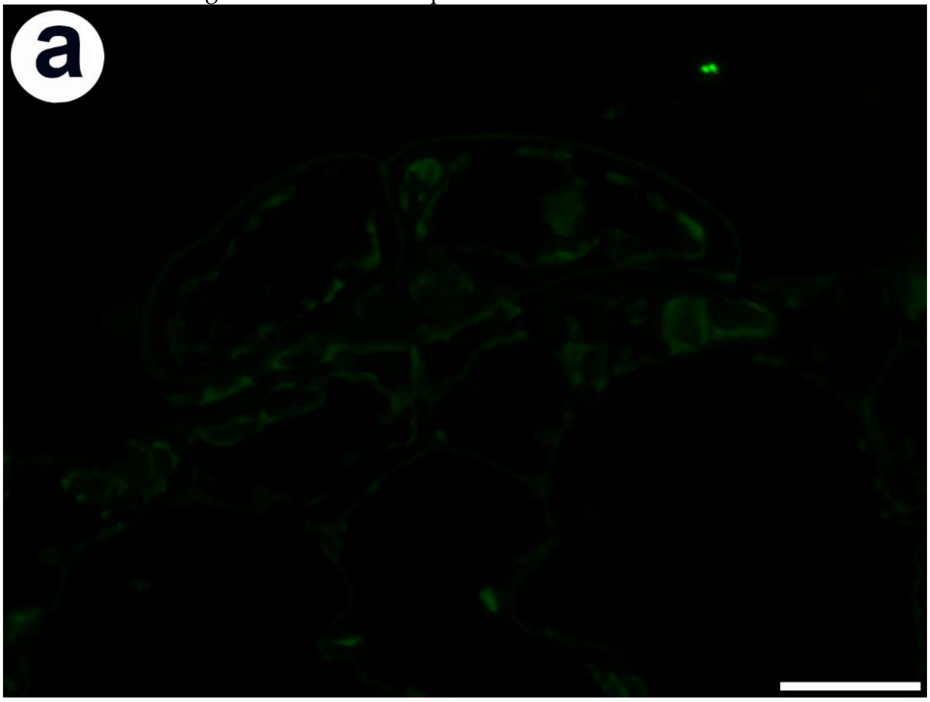

Supplement: Supplementary file 1 [file ijms-24-03358-s001.zip › ijms-2208777-supplementary.pdf]
